# Neuropsychiatric Symptoms in Patients with Idiopathic Normal Pressure Hydrocephalus

**DOI:** 10.3233/BEN-2009-0233

**Published:** 2009-12-07

**Authors:** Yumiko Kito, Hiroaki Kazui, Yoshihiko Kubo, Tetsuhiko Yoshida, Masahiko Takaya, Tamiki Wada, Keiko Nomura, Mamoru Hashimoto, Shingo Ohkawa, Hiroji Miyake, Masatsune Ishikawa, Masatoshi Takeda

**Affiliations:** ^1^PsychiatryDepartment of Integrated MedicineDivision of Internal MedicineOsaka University Graduate School of MedicineSuita-cityOsakaJapan; ^2^Department of PsychiatryJapan Labour Health and Welfare Organization Kansai Rousai HospitalAmagasaki-cityHyogoJapan; ^3^Department of PsychiatryNational Hospital Organization Osaka National HospitalChuo-kuOsaka-cityOsakaJapan; ^4^Institute for Aging Brain and Cognitive DisordersHyogo Brain and Heart Center at HimejiHimeji-cityHyogoJapan; ^5^Department of Psychiatry and NeuropathobiologyGraduate School of Medical SciencesKumamoto UniversityKumamoto-cityKumamotoJapan; ^6^Ohue HospitalAsagoshi-choAsagoshi-cityHyogoJapan; ^7^Nishinomiya Kyoritsu Neurosurgical HospitalNishinomiya-cityHyogoJapan; ^8^Department of NeurosurgeryKitano HospitalKita-kuOsaka-cityOsakaJapan; ^9^Department of NeurosurgeryRakuwakai Otowa HospitalYamashina-kuKyoto-cityKyotoJapan

**Keywords:** idiopathic normal pressure hydrocephalus, neuropsychiatric symptoms, apathy, Neuropsychiatric Inventory (NPI), Alzheimer's disease

## Abstract

*Objective:* To clarify the characteristics of neuropsychiatric symptoms in patients with idiopathic normal pressure hydrocephalus (iNPH).

*Methods:* Neuropsychiatric symptoms of 64 iNPH patients with mild triad symptoms from three kinds of hospitals were evaluated with the Neuropsychiatric Inventory (NPI) and compared with 126 patients with Alzheimer’s disease (AD).

*Results:* The most frequently observed neuropsychiatric symptom in the iNPH patients was apathy followed by anxiety and aggression. No symptom was more prevalent or more severe in iNPH than in AD. The severity of cognitive impairment was correlated with both aberrant motor activity and apathy.

*Conclusions:* Neuropsychiatric symptoms were mild in patients with iNPH and apathy was the most prevalent symptom. The correlation between neuropsychiatric symptoms and cognitive impairment in iNPH appears to arise from a common pathology in the frontal lobe.

## 1. Introduction

Normal pressure hydrocephalus (NPH) was first characterized in 1965 by Hakim and Adams as a progressive syndrome accompanied by the triad of gait disturbance, cognitive impairment, and urinary dys-

ISSN 0953-4180/09/$17.00 © 2009 – IOS Press and the authors. All rights reserved function [1]. NPH is generally classified into secondary NPH (sNPH), which is preceded by a disease such as subarachnoid hemorrhage, meningitis, and head trauma, and idiopathic NPH (iNPH), which has no causative antecedent disease. Recent improvements in methods for diagnosing and treating iNPH have resulted in increasing interest in this disease and in the publication of guidelines for its diagnosis and treatment [20, 32].

Although the characteristics of the triad symptoms have been recently clarified [7,42,47,51], the characteristics of neuropsychiatric symptoms in iNPH patients remain unclear. It is important to understand these characteristics because neuropsychiatric symptoms affect the quality of life of dementia patients [3,38] and increase the stress of caregivers [34]. A Swedish group evaluated the neuropsychiatric symptoms of iNPH patients and found some disorders, such as astheno-emotional syndrome [27,30]. However, these studies used small numbers of subjects, were biased toward one type of subject (those that visited a neurology department) and used a newly developed inventory [31] that did not fully cover the spectrum of neuropsychiatric symptoms.

In this study, we recruited 64 patients with iNPH from three kinds of institutions, a psychiatric clinic, two neurosurgical clinics, and a dementia center, and 126 patients with Alzheimer's disease (AD) who were age- and MMSE-comparable with the iNPH patients. We evaluated the neuropsychiatric symptoms in the patients with iNPH and AD by using the Neuropsychiatric Inventory (NPI) [10] and compared the neuropsychiatric symptoms between the two groups. The NPI is a comprehensive tool for assessing a wide variety of behavioral and psychiatric abnormalities in organic mental disorders including dementia that has been used in many studies in many countries [11,18,46]. We also assessed the relationship between the triad symptoms and the neuropsychiatric symptoms in 40 of the 64 iNPH patients and the change in neuropsychiatric symptoms in 10 of the 64 iNPH patients that underwent shunt operations.

## 2. Methods

This study was approved by the ethical committee of each institution and the study was carried out in accordance with the ethical standards of the 1964 Declaration of Helsinki. After a complete description of all procedure of this study, written informed consent was obtained from all subjects or their caregivers.

### *2.1. Subjects*

#### *2.1.1. Patients with iNPH*

This study was performed in four hospitals representing three types of institution: a dementia center, a psychiatric clinic, and two neurosurgical clinics. The Hyogo Institute for Aging Brain and Cognitive Disorders (HIABCD) is a dementia center and the site where this study began in January 1996. Patients meeting the following criteria were recruited consecutively from January 1996 to December 2003 at HIABCD. One author (H.K.) moved to the department of Neuropsychiatry of Osaka University Hospital, which is a psychiatric clinic for patients with psychiatric disorders, such as depression, schizophrenia, and anxiety disorder, as well as dementia. There, he recruited additional subjects from January 2004 to March 2008. Nishinomiya Kyouritsu Neurosurgical Hospital (N-neurosurgical clinic) and the department of Neurosurgery of Kitano Hospital (K-neurosurgical clinic) are neurosurgical clinics in general hospitals. These two clinics participated in this study from January 2004 to April 2006.

The criteria for probable iNPH included (1) age $>$ 60 years; (2) one or more of the triad of gait disturbance, cognitive impairment and urinary disturbance; (3) a magnetic resonance image (MRI) showing dilatation of the ventricular system and narrowed sulci at the convexity without severe cortical atrophy; (4) absence of diseases or conditions that might explain the clinical symptoms or radiological findings; (5) no history or evidence of conditions that might cause secondary NPH; (6) normal cerebrospinal fluid (CSF) pressure at lumbar puncture, and (7) improvement of symptoms after CSF tapping. We did not include patients with a negative CSF tap test because such patients rarely improve after shunt operations [12,55], and so are rarely recommended for shunt operations in our institutions. In the CSF tap test, lumbar puncture was performed with a thick needle (19 gauge or larger). CSF was removed until the total volume removed reached 30 ml or until CSF pressure reached 0 mm $H_2O$. The triad symptoms were evaluated with a rating scale, cognitive tests, such as the Mini-Mental State Examination (MMSE) [15], and tests for walking, such as the 3 m Up and Go test [44], before, 1 day after, and 1 week after CSF tapping. CSF tapping was judged to have improved symptoms if at least one symptom improved at either 1 day or 1 week after tapping. The exclusion criteria were (1) absence of reliable informants to assess NPI, (2) inability to obtain consent to this study, and (3) failure to interview the caregiver.

Table 1
Demographic and characteristics of the subjects with iNPH and AD

| | iNPH | | | | | P value | |
|---|---|---|---|---|---|---|---|
| Sex, age, type of test | Total | Psychiatric clinic | Neurosurgical clinics | Dementia center | AD | among iNPH | iNPH vs AD |
| Males/females | 38/26 | 9/12 | 16/3 | 13/11 | 41/85 | 0.020 | < 0.001 |
| Age | 74.9 ± 5.9 | 73.6 ± 7.0 | 75.0 ± 5.3 | 76.0 ± 5.1 | 74.3 ± 6.5 | 0.375 | 0.540 |
| MMSE | 20.6 ± 5.8 | 19.8 ± 5.8 | 22.9 ± 5.7 | 19.6 ± 5.6 | 20.6 ± 5.7 | 0.117 | 0.991 |
| Triad numbers of patients (percentages) | | | | | | | |
| gait disturbance | 54 (84.3) | 18 (85.7) | 19 (100) | 19 (79.2) | – | 0.105 | – |
| cognitive impairment | 56 (87.5) | 21 (100) | 15 (78.9) | 20 (83.3) | – | 0.080 | – |
| urinary disturbance | 50 (78.1) | 17 (81.0) | 18 (94.7) | 15 (71.4) | – | 0.036 | – |
| iNPHGS | | | | | | | |
| gait disturbance | 2.0 ± 0.5 | 1.8 ± 0.6 | 2.2 ± 0.4 | – | – | 0.015 | – |
| cognitive impairment | 2.3 ± 0.9 | 2.7 ± 0.6 | 1.9 ± 1.0 | – | – | 0.008 | – |
| urinary disturbance | 1.8 ± 1.0 | 1.7 ± 1.0 | 1.8 ± 1.0 | – | – | 0.480 | – |

MMSE = Mini-Mental State Examination.

The numbers of the patients from each kind of institution were similar (Table 1). Data were collected for only 16.6% of the patients in the N-neurosurgical clinic and for only 16.4% of the patients in the K-neurosurgical clinic because these clinics did not have qualified raters (geriatric psychiatrists or neurologists) on their staffs. The patients were interviewed by geriatric psychiatrists from the psychiatry clinic who made visits to the neurosurgical clinics. None of the patients at the other two institutions were excluded for this reason. Sixty-four iNPH patients fulfilled the criteria. The male/female ratio was significantly higher in the neurosurgical clinics than in the psychiatric clinic and the dementia center ($p = 0.007$, 0.04, respectively, post hoc Chi-square test). The ages and MMSE scores among the three kinds of institution were not significantly different (one-way ANOVA, $p = 0.38$, $p = 0.12$, respectively). Among the triad of symptoms, urinary disturbance was significantly more frequent in patients from the neurosurgical clinics than in those from the dementia center ($p = 0.01$, post hoc Fisher's exact probability test). No significant differences were observed in gait disturbance or cognitive impairment among the three kinds of institutions.

The only iNPH patients we could follow up on after the shunt operations were those who were from the psychiatric clinic. Ten of the 21 iNPH patients were given the shunt operations. The rest of the patients did not have shunt operations because of refusal of the patients or their family, diseases which interfered with the shunt operation, or advanced age. The mean age of the 10 patients (4 men and 6 women) was 72.3 ± 5.4 years and the mean MMSE score before the shunt operation was 23.1 ± 3.6. The mean iNPHGS scores for cognitive impairment, gait disturbance and urinary disturbance before the shunt operation were 2.5 ± 0.5, 1.8 ± 0.6, and 1.3 ± 0.9, respectively. These clinical data were not significantly different from those of all patients with iNPH ($p = 0.19$, 0.19, and 0.25, respectively). Seven patients received lumboperitoneal shunts, and three received ventriculoperitoneal shunts. After the shunt operations, one or more of the triad symptoms improved in all of the ten patients. The mean iNPHGS scores for cognitive impairment, gait disturbance and urinary disturbance after the shunt operations were 2.2 ± 0.4,1.5 ± 0.7, and 1.2 ± 1.3, respectively.

### 2.1.2. *Patients with AD*

AD subjects fulfilling the following criteria were consecutively recruited from the psychiatry clinic from September 2002 to March 2008. The inclusion criteria included (1) fulfillment of the criteria for probable AD of the National Institute of Neurological and Communicative Disorders and Stroke-Alzheimer's Disease and Related Disorders Association (NINCDS-ADRDA) [35] at the initial examination, or fulfilling the criteria for amnestic mild cognitive impairment [56] at the initial examination and fulfilling the criteria for probable AD of NINCDS-ADRDA, at a follow-up examination (2) absence of any other neurological diseases, (3) no evidence of focal brain lesions on MRI, and (4) an age of 60 or more at the first visit. The exclusion criteria were: (1) absence of reliable informants to assess NPI, (2) inability to obtain consent to this study, and (3) failure to interview the caregiver.

A total of 126 patients fulfilled the criteria (Table 1). The ages and the MMSE scores of the two groups of patients were not significantly different ($p = 0.54$, 0.99, respectively, Student's t test). The proportion of men was significantly higher in iNPH group ($p < 0.001$, Chi-square test).

### 2.2. Assessment of neuropsychiatric status

In the NPI, the caregivers were asked whether the patients had experienced any of the following 10 neuropsychiatric symptoms in the 30 days before the interview: delusions, hallucinations, depression, anxiety, agitation/aggression, disinhibition, euphoria, irritability, apathy, and aberrant motor activity. According to the criteria-based rating scheme, the severity of each manifestation was classified into grades 0 to 3 and the frequency of each manifestation was classified into grades 0 to 4, with 0 indicating absence of manifestation. The NPI composite score (severity x frequency) was calculated for each manifestation (range of possible scores, 0–12). Thus, the maximum total NPI score (for the 10 manifestations) is 120. The NPI interviews were conducted by geriatric psychiatrists or neurologists who knew psychiatric symptoms of dementia well. The neuropsychiatric symptoms were reevaluated three months after the shunt operations in ten iNPH patients from the psychiatric clinic.

### 2.3. Assessment of the triad of symptoms of iNPH

Forty of the 64 iNPH patients were from the psychiatric and neurosurgical clinics (Table 1). The severities of cognitive impairment, gait disturbance and urinary disturbance of these 40 patients were rated by using the iNPH Grading Scale (iNPHGS) [24]. The iNPHGS is a scale to separately assess the severity of each of the triad, and the score was rated based on observations by the physician and information from the patient or the caregivers. The score of each symptom ranges from 0 to 4, with higher scores indicating worse symptoms.

### 2.4. Statistical analyses

Differences in the prevalence of neuropsychiatric symptoms between iNPH and AD patients were evaluated with a Chi-square test. The difference in the NPI composite score between iNPH and AD groups was tested by Mann-Whitney U test. The prevalence of at least one of the 10 neuropsychiatric symptoms at the three kinds of institution was compared with a Chi-square test. The prevalence of each subscale at the three kinds of institution was compared with Fisher's exact probability test. The composite scores of the three kinds of institutions were compared with the Kruskal-Wallis test and the post hoc Scheffé test. The correlation between the iNPHGS scale and the NPI score was examined by using Spearman's rank correlation test.

Table 2
Prevalence (%) of neuropsychiatric symptoms in patients with iNPH and AD

| Symptoms | iNPH | AD | p value |
|---|---|---|---|
| At least 1 symptom | 73.4 | 91.3 | 0.001 |
| Delusion | 12.5 | 29.4 | 0.010 |
| Hallucination | 4.7 | 7.1 | 0.511 |
| Agitation | 17.2 | 45.2 | $<$ 0.001 |
| Depression | 14.1 | 36.5 | 0.003 |
| Anxiety | 25.0 | 34.1 | 0.199 |
| Euphoria | 7.8 | 4.0 | 0.262 |
| Apathy | 70.3 | 74.6 | 0.528 |
| Disinhibition | 12.5 | 8.7 | 0.258 |
| Irritability | 10.8 | 25.4 | 0.020 |
| Aberrant motor activity | 14.1 | 25.4 | 0.073 |

iNPH = idiopathic normal pressure hydrocephalus, AD = Alzheimer's disease

The composite scores before and after the shunt operations were compared with the Wilcoxon signed-rank test. The significance level was set at $p = 0.05$ because of the exploratory nature of this study. Analyses were performed using Statistica Ver.06J for Windows.

## 3. Results

### 3.1. Prevalence of neuropsychiatric symptoms in iNPH

Most of the iNPH patients (73.4%) showed at least one of the 10 neuropsychiatric symptoms included in the NPI subscale. The most prevalent symptom in iNPH was apathy (70.3%), followed by anxiety (25.0%) (Table 2). In all patients with aberrant motor activity, the aberrant motor activity was in the form of simple stereotyped behaviors such as hand clapping, swaying of body and rocking back and forth, or opening and closing doors without purposes.

### 3.2. Comparison of neuropsychiatric symptoms between iNPH and AD

Almost all of the AD patients (91.3%) had at least one of the neuropsychiatric symptoms. The prevalence of the neuropsychiatric symptoms in AD patients was significantly higher than that in the iNPH patients (Table 2) ($p = 0.001$). None of the symptoms was significantly more prevalent in the iNPH group than in the AD group, although delusion, agitation, depression, and irritability were less common in the iNPH group.

The mean NPI total score in the iNPH group (7.5 $\pm$ 11.4) was significantly lower than the score in the AD group (12.30 $\pm$ 11.1) ($p = 0.0001$). The AD group

Table 3
Prevalence (%) of neuropsychiatric symptoms in patients with iNPH at the three institutions

| Symptoms | Psychiatric clinic | Neurosurgical clinics | Dementia center | p value |
|---|---|---|---|---|
| At least 1 symptom | 85.7 | 52.6 | 79.2 | 0.044 |
| Delusion | 14.3 | 5.3 | 16.7 | 0.573 |
| Hallucination | 9.5 | 5.3 | 0 | 0.295 |
| Agitation | 19.0 | 10.5 | 20.8 | 0.707 |
| Depression | 14.3 | 15.8 | 12.5 | 0.953 |
| Anxiety | 38.1 | 26.3 | 12.5 | 0.049 |
| Euphoria | 0 | 15.8 | 8.3 | 0.214 |
| Apathy | 76.2 | 52.6 | 79.2 | 0.129 |
| Disinhibition | 4.8 | 15.8 | 16.7 | 0.423 |
| Irritability | 0 | 15.8 | 16.7 | 0.177 |
| Aberrant motor activity | 19 | 10.5 | 12.5 | 0.739 |

iNPH = idiopathic normal pressure hydrocephalus

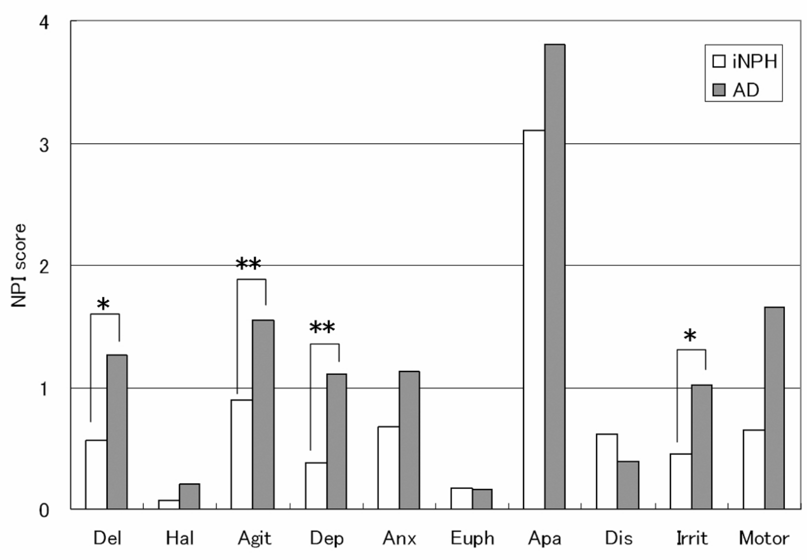

Fig. 1. Composite scores (frequency x severity) for neuropsychiatric symptoms in patients with iNPH and AD. * $p < 0.05$, ** $p < 0.01$. iNPH = idiopathic normal pressure hydrocephalus, AD = Alzheimer's disease, Del = delusion, Hal = hallucination, Agit = agitation, Dep = depression, Anx = anxiety, Euph = euphoria, Apa = apathy, Dis = disinhibition, Irrit = irritability, Motor = aberrant motor activity.

had significantly higher scores for delusion ($p = 0.01$), agitation ($p = 0.0004$), depression ($p = 0.0017$), and irritability ($p = 0.02$). The composite score of the iNPH group was not higher than that of the AD group in any of the domains (Fig. 1).

### 3.3. Comparison of neuropsychiatric symptoms among three kinds of institutions

The prevalences of neuropsychiatric symptoms were 85.7% in the psychiatry clinic, 52.6% in the neurosurgery clinics, and 79.2% in the dementia center (Table 3). A post-hoc Chi-square test revealed that the neuropsychiatric symptoms were significantly more prevalent in the psychiatry clinic than in the neurosurgery clinics ($p = 0.02$), however there were no significant differences between the neurosurgery clinics and the dementia center, or between the psychiatry clinic and the dementia center ($p = 0.07$ and $p = 0.57$, respectively). There were no significant differences in the prevalence of each subscale among the institutions other than that of anxiety, which was higher in the psychiatry clinic than in the dementia center ($p = 0.046$).

The mean NPI total scores were 5.3 ± 3.8 (0–11) in the psychiatry clinic, 4.8 ± 9.7 (0–41) in the neurosurgery clinics, and 11.7 ± 16.4 (0–59) in the dementia center. There were no significant differences among the three institutions. The scores of all but one of the subscales were not significantly different. The exception was the apathy score, which was significantly higher

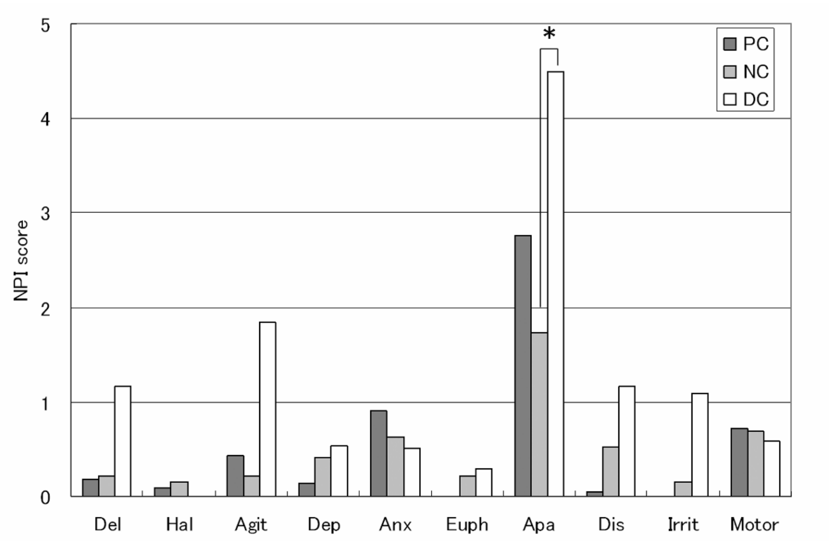

Fig. 2. Composite scores (frequency x severity) for neuropsychiatric symptoms in patients with iNPH at three institutions. *p < 0.05. PC = the psychiatry clinic, NC = the neurosurgery clinics; DC = the dementia center, Del = delusion, Hal = hallucination, Agit = agitation, Dep = depression, Anx = anxiety, Euph = euphoria, Apa = apathy, Dis = disinhibition, Irrit = irritability, Motor = aberrant motor activity.

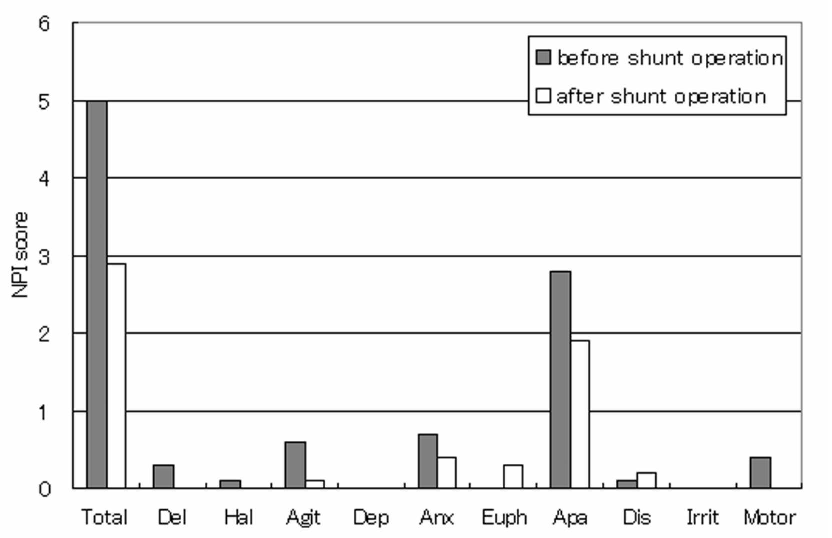

Fig. 3. Composite scores (frequency x severity) for neuropsychiatric symptoms in patients with iNPH before and after a shunt operation. iNPH = idiopathic normal pressure hydrocephalus, Total = NPI total score, Del = delusion, Hal = hallucination, Agit = agitation, Dep = depression, Anx = anxiety, Euph = euphoria, Apa = apathy, Dis = disinhibition, Irrit = irritability, Motor = aberrant motor activity.

in the dementia center than in the neurosurgery clinics ($p = 0.01$) (Fig. 2).

### *3.4. Association between neuropsychiatric symptoms and triad symptoms*

The mean iNPHGS scores of the 40 patients from the psychiatric and neurosurgical institutions were 2.0 ± 0.6 for gait disturbance, 2.3 ±0.9 for cognitive impairment, and 1.8 ± 1.0 for urinary disturbance (Table 1). The NPI scores of aberrant motor activity were significantly correlated with the iNPHGS scores of cognitive impairment ($r = 0.49$, $p < 0.05$) and urinary disturbance ($r = 0.36$, $p < 0.05$). The apathy scores were significantly correlated with the scores of cognitive impairment ($r = 0.42$, $p < 0.05$). The gait scores of iNPHGS had no correlation with the NPI scores of any subscales.

### 3.5. *Assessment of the neuropsychiatric symptoms after shunt operation*

The mean NPI total scores before and after the shunt operations were 5.0 ± 3.6 (0–11) and 2.9 ± 2.8 (0–9) respectively, and there were no significant differences ($p = 0.13$) (Fig. 3). After the shunt operation, all of the score for the NPI subscales except for the score for euphoria and disinhibition decreased, but none of the changes were statistically significant.

## 4. Discussion

In the present study, we examined the neuropsychiatric and behavioral symptoms in 64 iNPH patients. The institution to which an iNPH patient is referred normally depends on his or her symptoms. For example, among the triad symptoms, the prevalences of cognitive impairment and urinary incontinence in iNPH patients referred to a department of neurosurgery (98% and 83%, respectively) [23] were higher than those in patients referred to a multidisciplinary memory clinic (69%, and 54%) [5]. Similarly, the neuropsychiatric symptoms of patients with frontotemporal lobar degeneration were found to affect the choice of institution by the caregivers [19]. Therefore, to avoid such biases, and thus obtain more reliable prevalences of the different neuropsychiatric symptoms, we selected patients from three kinds of institutions, a psychiatric clinic, two neurosurgical clinics and a dementia center.

In the present study, the percent of the iNPH patients who showed at least one of the 10 neuropsychiatric symptoms included in the NPI subscale was 73.4%, and the mean NPI total score was 7.5, which were significantly lower than those in AD patients. Apathy was the most frequent symptom and was present in 70% of the iNPH subjects, but the degree of apathy was not severe in patients with iNPH in this study. For comparison, the prevalence of neuropsychiatric symptoms in normal elderly people was reported to be at most 27% and the prevalence of apathy was reported to be 4.8% [17]. Thus, the prevalences of neuropsychiatric symptoms and apathy in iNPH patients were higher than those in the normal elderly. Apathy has been considered as a prominent symptom in NPH in many studies and reviews [14,37,45]. However, these studies did not systematically assess the neuropsychiatric symptoms in NPH. A Swedish group attempted to clarify the neuropsychiatric symptoms systematically in a number of iNPH patients [27,30]. However, they used a specific and original method [31] that evaluated the neuropsychiatric symptoms of only five domains of psychiatric disorders: somnolence-sopor-coma disorder (SSCD), astheno-emotional syndrome (AE), emotional-motivational syndrome (EM), confusional syndrome, and Korsakoff's amnesia. In one study assessing 26 iNPH patients, AE was seen in 70% of the patients, EM in 40%, and SSCD in 35% [27]. Another study found AE in 100% of the patients, SSCD in 40%, and EM in 0% [30]. Apathy is a component of SSCD and AE, two of the most frequent disorders found in the Swedish studies. Thus, our finding that apathy is the most frequent symptom in iNPH is consistent with the findings of the Swedish studies. The present study is the first to compare the severity of apathy in patients with iNPH and patients with another form of dementia (AD). We found that the mean NPI apathy scores of the iNPH and AD patients were equivalent. However, the NPI apathy score is known to be significantly higher in patients with frontotemporal dementia (FTD) and vascular dementia than in those with AD [13,16, 28]. Therefore, apathy may be milder in iNPH patients than that in patients with FTD and vascular dementia. Another factor contributing to the mild apathy levels in our iNPH patients might be the early stage at which they were recruited, as reflected by their less severe MMSE and iNPHGS scores (approximately 20 and 2, respectively).

Anxiety, agitation and stereotyped behavior were observed in 25%, 17%, and 14% of patients with iNPH respectively. The present study is the first to clearly detect these behaviors in iNPH patients, possibly because it is the first to use the NPI, which is specifically designed to assess them. The method [31] used in the previous studies [27,30] had no items for detecting anxiety and stereotyped behavior. It also failed to measure agitation itself correctly, although agitation might be included in AE which combined some neuropsychiatric symptoms.

The neuropsychiatric symptoms in patients with iNPH can be attributed to brain damage. Apathy has been suggested to have associations with brain areas such as the anterior cingulate cortex (ACC), and thalamus [9,26,33], which constitute the frontal-subcortical circuits. Because hypoperfusion in the ACC and thalamus was found in iNPH patients [22,40,52], apathy in iNPH could arise from dysfunction in these regions. Since brain functional imaging studies in iNPH patients have shown a functional decline in the orbitofrontal area as well as in the ACC [22,40], the involvement of the orbitofrontal area could induce anxiety, aggression, and stereotyped behavior [2,6,8,36]. The correlations between some neuropsychiatric symptoms and the cognitive impairment and urinary dysfunction in the present study may also indicate frontal dysfunction, which has previously been associated with these dysfunctions in iNPH patients [42,48]. The improvements in the neuropsychiatric symptoms and triad symptoms in our patients that underwent shunt operations may be due to improved oxygen metabolism or cerebral perfusion in the frontal cortex and thalamus, which are known to occur in iNPH patients following shunt operations [39, 54].

In this study, the composite scores for some of the NPI subsets, such as delusion, agitation, irritability, and depression, were lower in the iNPH patients than in the AD patients, and no symptoms were more prevalent or more severe in the iNPH patients than in the AD patients. This may be because the two groups have different patterns of brain dysfunction. The brain dysfunction in iNPH is mainly in the frontal cortex, whereas the major dysfunction in AD arises from the medial temporal cortex, including the amygdala [21], which has an important role in regulating emotion. According to a recent fMRI study in patients with AD [57], amygdala activity was found to be correlated with the severity of neuropsychiatric symptoms, such as irritability and agitation. The absence of the amygdala involvement in an early stage of iNPH could result in neuropsychiatric symptoms that are milder than those that occur in AD. Another reason might be that AD patients often have an asymmetry of brain function or brain volume [43, 53], while brain laterality has not yet been reported in iNPH patients. In brain functional imaging studies in AD [25,29,41,49], delusion, agitation, irritability, and depression are reported to associate with right dominant brain dysfunction. The absence of brain laterality would result in less severe neuropsychiatric symptoms than are observed in AD patients.

The present study has several limitations. First, only 16% of the patients from the two neurosurgical clinics were included in this study, which might have caused a bias in the patients from these clinics. Second, the demographic data between the iNPH and AD groups was not fully matched in this study. The percent of women was significantly higher in the AD subjects, and we did not take into consideration the duration of the diseases. Some studies of dementia reported that both sex and duration influence psychiatric symptoms [50, 58]. Third, we can not rule out the possibility that our screening of iNPH patients did not remove patients with comorbidity of AD, which has been observed in some iNPH patients [4]. These issues should be taken into consideration before the findings are generalized.

In this present study, we have elucidated the neuropsychiatric features of patients with relatively mild stages of iNPH. Future studies that include patients with more severe stages of iNPH are needed to more precisely recognize the pattern of neuropsychiatric symptoms of iNPH patients.
